# 

**Published:** 1880-09-20

**Authors:** 


### EDITORIAL AND MISCELLANEOUS.

☞Please *Send up your dues* and renew your subscription.

*Subscription receipted* will appear in our next.

*The Southern Medical College* will open its second session on the 13th of October next. A large class is anticipated.

*Medical Books.*—See advertisement of LYNCH & THORNTON, clever gentlemen and enterprising book merchants, Atlanta, Ga.

*Parke, Davis & Co.*—This enterprising establishment is still bringing out new and valuable agents. See their new advertisement in this issue.

*Correction.*—The article in our August issue on gelseminum, credited to London Med. Journal, Dr. Hobbs, ought to have been credited to Louisville Med. News, Dr. A. G. Hobbs.

*Lectures on Lithotrity.*—Prof. Reuben A. Vance, of Cincinnati, has engaged to deliver a series of lectures on the subject of "Modern Lithotrity to the Class of the Southern Medical College at the ensuing Session.

*Fluid Extract of Malt.*—We have a sample bottle of this article as prepared by Messrs. Merrell, Thorpe & Loyd, Cincinnati. It is neatly prepared, is palatable to the taste and is evidently an excellent preparation. See their advertisement.

*Beef Cod-Liver Oil and Pepsin.*—See the advertisement of this preparation. The combination is certainly a happy one. It is prepared by the Brown Chemical Co., Baltimore, Md., sole manufacturers of Powel's Prepared Chemicals.

*Typhoid Fever.*—This disease is prevailing in many localities the present season, and has proven unusually obstinate and protracted. Practical articles on this disease or on typho-malarial fever would be gladly received by the Editors of this Journal.

*Popular Science Monthly.*—This very instructive and interesting monthly we are glad to number among our exchanges. It furnishes most valuable reading to the intelligent practitioner and to men of scientific taste especially. It is published by the enterprising and popular establishment of D. Appleton & Co., New York.

*Liebig Laboratory and Chemical Works.*—We are in receipt of beautiful samples of "Coca Beef Tonic" and "Genuine Extract of Witch Hazel," prepared by the above enterprising establishment, which has both a London and New York house. These are valuable preparations neatly and tastefully put up. The sole agents in this country for their sale are J. L. Berg & Co., 87 William Street, New York.
See their advertisement in this Journal.

*College Fees.*—We have received announcements from the following Colleges: Medical College of Ohio, fees $75; Cincinnati College of Medicine, fees $75; Detroit Medical College, fees $75; Hospital College of Medicine, Louisville, fees $75; University of Louisville, fees $75; Medical College of Georgia, at Augusta, fees $75; New Orleans Medical College, fees $140; University of Pennsylvania, fees $140; Bellevue Medical College, fees $140; SOUTHERN MEDICAL COLLEGE, Atlanta, fees $75; Atlanta Medical College, fees $75.

*A New Hypodermic Syringe.*—F. C. Otto & Sons have manufactured a new hypodermic syringe of celluloid. Every part of the syringe is made of celluloid, except the needles. The barrel is as transparent as glass, and will not break by blows or falling.

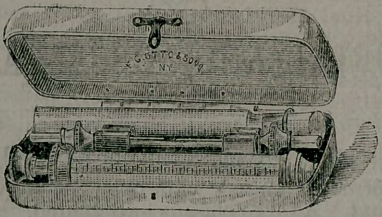

The cut above will give an idea of the syringe. It is neat and durable and supplies a want, and one our friends will appreciate. Price, $4 00 by mail. Send to F. C. Otto & Sons, 64 Chatham Street New York.

*A New School Physiology*, by Richard J. Dungleson, A.M., M.D., editor of "Dungleson's Medical Dictionary," "History of Medicine," Secretary of the Academy of Medicine, etc. Illustrated with one hundred and seventeen engravings; 314 pages.—Porter & Coates, Philadelphia, 1880.

As a member of the Board of Commissioners of the Public Schools of Atlanta, we have examined this work with special care, having an eye to the best books for the pupils, and hesitate not to say it is the best work as a School Physiology we have seen.

The time has come when the importance of physiological study in the common schools is acknowledged and appreciated. The recent agitation of sanitary matters in this country, the appointment of Boards of health, and the interest in public health excited thereby has given a decided impetus to all classes of studies having reference to the human body and to the laws of hygiene.

Apart from these considerations, the subject is one of absorbing interest in itself, embracing, as it does, much of comparative anatomy, chemistry and natural history, and covers a wide and interesting field, and is calculated to lead the mind of the student into important and elevated conceptions of both the physical and mental man, and of the evidences of wisdom and design in the works of creation.

The book is neatly gotten up, the print is plain, the style good, the illustrations well drawn, and though sufficiently comprehensive as to include all the important topics of the science, is yet free from unnecessary detail, and of a size admirably suited as a text book for common schools. We trust the publishers will send for inspection copies of this work to the superintendents of Public Schools throughout the country.

